# The spatio-temporal mapping of epileptic networks: Combination of EEG–fMRI and EEG source imaging

**DOI:** 10.1016/j.neuroimage.2009.01.070

**Published:** 2009-07-01

**Authors:** S. Vulliemoz, R. Thornton, R. Rodionov, D.W. Carmichael, M. Guye, S. Lhatoo, A.W. McEvoy, L. Spinelli, C.M. Michel, J.S. Duncan, L. Lemieux

**Affiliations:** a National Society for Epilepsy MRI Unit, Department of Clinical and Experimental Epilepsy UCL Institute of Neurology and National Hospital for Neurology and Neurosurgery, Queen Square, London, UK; b Presurgical Evaluation for Epilepsy Unit, Neurology Department, University Hospital and University of Geneva, Switzerland; c Functional Brain Mapping Laboratory, Neurology Department, University Hospital and University of Geneva, Switzerland; d CNRS UMR 6612 and Service de Neurophysiologie Clinique, Faculté de Médecine and CHU Timone, Marseille, France; e Department of Neurology, North Bristol NHS Trust, Frenchay Hospital, Frenchay Road, Bristol, UK

## Abstract

Simultaneous EEG–fMRI acquisitions in patients with epilepsy often reveal distributed patterns of Blood Oxygen Level Dependant (BOLD) change correlated with epileptiform discharges. We investigated if electrical source imaging (ESI) performed on the interictal epileptiform discharges (IED) acquired during fMRI acquisition could be used to study the dynamics of the networks identified by the BOLD effect, thereby avoiding the limitations of combining results from separate recordings.

Nine selected patients (13 IED types identified) with focal epilepsy underwent EEG–fMRI. Statistical analysis was performed using SPM5 to create BOLD maps. ESI was performed on the IED recorded during fMRI acquisition using a realistic head model (SMAC) and a distributed linear inverse solution (LAURA).

ESI could not be performed in one case. In 10/12 remaining studies, ESI at IED onset (ESIo) was anatomically close to one BOLD cluster. Interestingly, ESIo was closest to the positive BOLD cluster with maximal statistical significance in only 4/12 cases and closest to negative BOLD responses in 4/12 cases. Very small BOLD clusters could also have clinical relevance in some cases. ESI at later time frame (ESIp) showed propagation to remote sources co-localised with other BOLD clusters in half of cases. In concordant cases, the distance between maxima of ESI and the closest EEG–fMRI cluster was less than 33 mm, in agreement with previous studies.

We conclude that simultaneous ESI and EEG–fMRI analysis may be able to distinguish areas of BOLD response related to initiation of IED from propagation areas. This combination provides new opportunities for investigating epileptic networks.

## Introduction

In patients with epilepsy, non-invasive imaging techniques can offer precious information regarding the localisation and dynamics of epileptic activity. In the particular case of patients with pharmaco-resistant focal epilepsy, this information can be used to select patients for surgical treatment or to identify target regions for intracranial EEG recording.

EEG-correlated functional Magnetic Resonance Imaging (EEG–fMRI) is a rapidly developing non-invasive imaging technique that can map regional changes in the cerebral oxygenation and blood flow (BOLD signal (Ogawa et al., 1990)) that are time-locked to Interictal Epileptiform Discharges (IED) identified on the simultaneously recorded EEG. In its current form, EEG–fMRI can reveal maps showing regions of BOLD change correlated to IEDs. Comparison of the EEG–fMRI maps with electro-clinical data have shown significant BOLD increases and decreases both near to and distant from the presumed the irritative zone (regions involved in IED generation) (Aghakhani et al., 2004; Kobayashi et al., 2006a; Salek-Haddadi et al., 2006; Jacobs et al., 2007). Some authors have focused their attention on regions with the most significant BOLD response to determine concordance with electro-clinical and radiological findings (Salek-Haddadi et al., 2006) and others considered only positive BOLD response to assess concordance (Krakow et al., 1999; Lazeyras et al., 2000; Al-Asmi et al., 2003). The underlying neurophysiological mechanism of negative BOLD changes particularly when correlated to IEDs remains unclear and may reflect decreases in neuronal activity and blood flow (Shmuel et al., 2002; Parkes et al., 2004; Stefanovic et al., 2005; Kobayashi et al., 2006b; Carmichael et al., 2008).

Electric source imaging (ESI) is the reconstruction of the three-dimensional brain electrical activity derived from scalp EEG data, relying on various mathematical algorithms and constraining neurophysiological hypotheses (see Michel et al., 2004 for a review). This technique benefits from a very high temporal resolution corresponding to the EEG sampling frequency and from the fact that EEG reflects neural activity (local synchrony) more directly than fMRI. However, the use of ESI for events resulting from widespread, deep-seated or multi-regional brain activity remains problematic.

A few studies have evaluated the concordance between various ESI methodologies (single or multiple equivalent dipole, distributed solutions, Bayesian approaches) and EEG–fMRI results in focal epilepsy (Seeck et al., 1998; Lemieux et al., 2001; Bagshaw et al., 2006; Benar et al., 2006; Boor et al., 2007; Grova et al., 2008) but the ESI and EEG–fMRI analyses were not performed on simultaneously acquired datasets. All studies found a good degree of spatial concordance between ESI results and EEG–fMRI but also stressed the intrinsically different underlying neurophysiological mechanisms for each measurement: ESI directly images synchronised synaptic activity whereas fMRI measures a resulting mixed contribution of focal metabolic and perfusion changes. In the present study we combined ESI and EEG–fMRI in patients with focal epilepsy in order to address three questions:

1) Can one discriminate between BOLD responses coupled to onset vs. propagation of IED with the help of the temporal resolution of ESI?
2) Is the cluster with the most significant positive BOLD response a good localiser of the region of IED onset (as identified using ESI)?
3) Do negative BOLD changes have localising value with respect to the zone of IED onset or are they only a reflection of distant upstream or downstream effects?

## Methods

### Patients and electro-clinical data

Patients with refractory focal epilepsy undergoing pre-surgical assessment were selected from our database of EEG–fMRI at 3 T since January 2004 according to the following criteria: 1) intra-MRI EEG recording with 32 electrodes or more; 2) presence of spikes, spike-waves or sharp waves on the intra-MRI EEG. Epileptic transients in the form of short runs of low amplitude high frequency poly-spikes were not considered as they have poor signal-to-noise ratio for ESI and are best modelled as blocks for the EEG–fMRI analysis (Bagshaw et al., 2005); 3) the presence of a significant IED-related BOLD response (*p* < 0.05, Family-wise error correction for multiple voxel comparisons).

Nine patients were thus identified fulfilling the criteria (one EEG–fMRI recording each). In total, 12 types of IED were identified and used for analysis, resulting in 12 spike-type specific analyses. Clinical, electrophysiological and imaging data of the patients are given in Table 1. All patients had cryptogenic focal epilepsy except for patient 4 who had focal cortical dysplasia in the left mesial occipital cortex. The study was approved by the committee of the UCL Institute of Neurology and UCL Hospitals. Written informed consent was obtained from all patients.

Three patients underwent intracranial EEG investigations: in patient 1, a subdural grid with 6 × 8 contacts was placed over the right temporal cortex. One additional subdural strip electrode (1 × 8 contacts) was placed over the temporo-parietal cortex and 2 depth electrodes (6 contacts) targeted the right amygdala and anterior hippocampus. In patient 5, 8 depth electrodes with 15 contacts were placed stereotactically in the right hemisphere (6 orthogonal electrodes (medial/lateral contacts): i. (orbito-frontal antero-medial/orbito-frontal lateral), ii. (anterior cingulate gyrus/dorso-lateral prefrontal) iii. (insula/fronto-opercular (pars opercularis)), iv. (insula/fronto-opercular (pars orbicularis)), v. (supplementary motor area/dorsal premotor cortex), vi. (amygdala/middle temporal gyrus); 2 oblique electrodes: i. (orbito-frontal postero-medial/fronto-polar), ii. (dorsomedian thalamus/dorso-lateral-premotor) and 1 electrode was placed in the left hemisphere: (orthogonal electrode: anterior cingulate gyrus/dorso-lateral prefrontal). In patient 8, 4 electrodes (15 contacts) were implanted in the right hemisphere (3 orthogonal electrodes: i. (anterior cingulate gyrus/dorsolateral prefrontal), ii. (supplementary motor cortex/dorsal premotor cortex), iii. (paracentral lobule/precentral lateral); 1 oblique electrode: (postcentral/medial superior parietal lobule).

### EEG–fMRI acquisition

Prior to scanning, EEG was recorded for 15 min with eyes closed outside the scanner using MR-compatible systems EEG cap (Brain Products, Munich, Germany). A 32- or 64-electrode EEG cap was used according to the 10–20 or 10–10 electrode position convention. All patients underwent EEG–fMRI on a 3 T Signa Excite HDX scanner (GE Medical Systems, Milwaukee). They were asked to lie still in the scanner with their eyes closed and no specific instruction regarding vigilance was given. EEG was recorded continuously during fMRI along with a synchronisation signal from the scanner. ECG was recorded with a single lead. Each patient underwent 2 or 3 20-minute blocks of EEG–fMRI acquisition. Each fMRI dataset consisted of 404 T2⁎-weighted single-shot gradient-echo echo-planar images (EPI; TE/TR 30/3000 ms, flip angle 90, FOV 24 × 24 cm^2^, 43 interleaved slices with 3 mm thickness). For the purpose of anatomical localisation and EEG source localisation, we also acquired one volumetric 3D T1-weighted image including nasion and inion (resampled offline to 1 × 1 × 1 mm^3^ voxels for comparison with isotropic images created with the head model for ESI) as well as one high resolution EPI image.

### EEG processing and interpretation

Gradient artefacts from the scanner and pulse-related artefacts were removed offline from the EEG trace recorded during scanning using average artefact subtraction methods described elsewhere (Allen et al., 1998; Allen et al., 2000). The artefact-corrected EEG is unfiltered and contains frequencies from DC to 250 Hz (downsampled from the recorded 5000 Hz in the course of the artefact correction). Filtering of the EEG signals was needed to identify IED. Second order Butterworth Low and High pass filters (Low pass 35 Hz, High pass 0.3 Hz) with − 12 dB/octave roll-off were used. They were computed linearly with two passes (one forward and one backward), eliminating the phase shift, and with poles calculated each time to the desired cut-off frequency. IED were then averaged. Interictal Epileptiform Discharges (IED) were identified and marked consensually by two experienced electro-encephalographers (SV, RT) (Salek-Haddadi et al., 2006). Selection and categorisation of IED was based on a) location and morphology of the individual IED and b) voltage map topography. In one patient (patient 5) with very frequent IED (> 50/min) where we used a spike detection software algorithm based on spatio-temporal correlation (BESA, MEGIS software GmbH, Penzberg, Germany) and subsequently reviewed the detected IED to correct for false positive markers.

In four patients a total of 7 IED types were found not to be associated with significant BOLD changes (see below for description of fMRI analysis) and therefore were not considered further (see Table 1). In patient 1 three sets of IED were identifiable from the scalp EEG (case 1a: right mid-temporal, case 1b: right posterior temporal; case 1c: right occipito-temporal) and were considered separately for fMRI modelling and ESI in an initial analysis, 1c alone being associated with significant BOLD changes. Since their sites of origin were presumably very close to each other we performed an additional fMRI analysis by merging all three types of event, giving a grand total of 13 analyses.

### fMRI processing and statistical analysis

After discarding the first four image volumes, necessary to reach T1 stability, the fMRI time-series were realigned and spatially smoothed with a cubic Gaussian kernel of 8 mm full width at half maximum. fMRI time-series data were then analysed using a General Linear Model (GLM) implemented in the SPM5 software package (www.fil.ion.ucl.ac.uk/SPM) to determine the presence of regional IED-related changes of the BOLD signal. For each patient, a separate set of regressors in the same GLM was formed for each type of epileptiform discharge identified in the EEG, allowing the identification of specific BOLD effects. For this purpose, discharges were modelled as zero-duration events (unit impulse, or ‘delta’, functions) and convolved with the canonical Haemodynamic Response Function (HRF) as well as its temporal and dispersion derivatives, to account for deviations from the canonical time course. This resulted in three regressors for each event type (Friston et al., 1998). Motion-related effects were included in the general linear model (GLM) in the form of 24 regressors representing the Volterra expansion of the 6 realignment parameters (Friston et al., 1996), plus combinations of Heaviside step functions accounting for large motion effects (‘scan nulling’ regressors with 0.5 mm threshold) (Lemieux et al., 2007). An additional set of cardiac confound regressors was included to account for pulse-related signal changes (Liston et al., 2006). SPM-F contrasts were used across the three regressors corresponding to each event type to generate *F*-maps with a significance threshold of *p* < 0.05 corrected for multiple comparisons (family-wise error). The sign of the BOLD response at a given cluster was assessed by plotting the fitted response. In discordant cases (see below for definition), additional uncorrected *F*-maps (*p* < 0.001, uncorrected, cluster size > 10 voxels) were visually inspected to check for supra threshold changes in the suspected epileptogenic zone. Maps of significant BOLD responses were overlaid onto high-resolution EPI images and T1-weighted volumetric images after co-registration to the EPI images, allowing for anatomical localisation (Ashburner and Friston 1997).

### EEG source imaging (ESI)

The IED used for fMRI modelling were analysed with ESI. IED were then averaged and channels containing artefacts were interpolated. We used the SMAC head model which is based on a 3-shell spherical realistic head model and the patient's individual MRI (Spinelli et al., 2000). The source space was limited to the grey matter, segmented using SPM5 and a standard localisation of the electrodes according to the 10–20 system was assumed. The distributed inverse solution LAURA was used to solve the inverse problem (Grave de Peralta Menendez et al., 2001). The analysis was performed using Cartool software (http://brainmapping.unige.ch/Cartool.htm) for spike averaging and display of inverse solution onto the anatomical T1-weighted MR image. We localised IED onset (ESIo) as the maximum source activity occurring at half maximum of the first rising phase of the global field power of the averaged IED. This was previously reported to be the best estimation of IED onset (Lantz et al., 2003b). ESI of propagation areas of IED (ESIp) were defined as maximum source activity around the first or second GFP maximum that involved a different cortical region than ESIo.

### Assessment of concordance of BOLD activation maps and ESI results

F-maps (with FWE corrected threshold) showing BOLD signal changes and ESI results, were overlaid on T1-weighted anatomical MR images. We then selected the anatomically concordant BOLD clusters (i.e. located in the same lobe as the ESI-derived source) whose maximum were closest to ESIo and ESIp, respectively. When visual anatomical concordance was found, the Euclidian distance between ESIo/ESIp maximum and the maximum of the closest BOLD cluster was measured using the MRIcro (www.sph.sc.edu/comd/rorden/mricro.html). ESI and BOLD clusters were considered discordant if in different lobes and separated by a major anatomical fissure or sulcus (sylvian or interhemispheric fissure), so that neighbouring frontal/parietal or parietal/temporal regions for instance were not considered discordant a priori although located in different lobes. ESI and BOLD cluster with lateral vs. mesial temporal localisation were also considered discordant. In addition the localisation of the BOLD cluster containing the global maximal positive response was identified for spatial comparison with ESIo and ESIp.

### Assessment of concordance with intracranial EEG results

When available, results of intracranial EEG with depth or subdural electrodes were compared with our results. The position of the intracranial electrode contacts was assessed visually on MRI T1-weighted images acquired after the electrode implantation. The anatomical position of contacts inside the irritative zone was compared to the ESI and EEG–fMRI results.

## Results

ESI and BOLD results are summarised in Table 2.

### Concordance at IED onset (ESIo)

In 1/13 analyses with a significant BOLD response, the ESI and scalp topography did not allow reliable source localisation and this study was discarded (patient 6b). In 9/12 remaining IED studies, we found good anatomical concordance between ESIo and one cluster of BOLD response, which also matched other electro-clinical localisation. In 2 studies with inter-hemispheric source activity (patient 5, patient 8b), ESI showed bilateral mesial frontal localisation with maximum activity in the hemisphere contra-lateral to the BOLD maximum which itself was concordant with the electro-clinical localisation; according to our strict definition of concordance based on the localisation of the maximum of the ESI solution these results must be labelled as discordant. In 2 other studies of temporal IED (studies 1a and 4), discordance corresponded to large separation within a lobe (mesial vs. lateral temporal lobe in study 1a; temporo-parietal vs. lateral anterior temporal in study 4b).

In 4/12 studies, the BOLD cluster containing the global statistical maximum (and corresponding to BOLD increase) was the closest to ESIo. In 4/12 studies the cluster closest to ESIo corresponded to a negative BOLD change. In 2 of these cases, the closest positive BOLD cluster was in the same lobe as ESIo but more distant than the cluster corresponding to BOLD decrease. In the two other cases, there were only negative BOLD changes.

The mean peak-to-peak Euclidian distance for the concordant cases was 23 ± 9 mm (range: 6–33 mm). When including the two cases with frontal mesial ESIo lateralised to the hemisphere contralateral to electro-clinical localisation, the distance values were 20 ± 10 mm (6–33 mm). In these two cases, the maximum voxel was projected across the midline for distance measurement. Distance was not measured in the remaining 2 discordant cases (patient 1: mesial vs. lateral temporal lobe; patient 4b: temporal pole vs. temporo-parietal junction).

### Concordance in IED propagation areas (ESIp)

In 6/12 IED studies, ESIp showed good concordance with one BOLD cluster that was positive in each case. The Euclidian distance for these concordant cases was 15 ± 7 mm (6–25 mm). In the 6 other cases, visual inspection of the uncorrected *F*-maps showed concordant uncorrected positive BOLD clusters in 2 cases but no concordant BOLD in the remaining cases.

### Comparison with intracranial EEG results

In patient 1, subdural grid electrodes recorded right lateral mid-temporal and lateral posterior temporal IED confirming our ESIo findings of separate temporal IED (Table 1). The largest and most diffuse of these lateral temporal IED showed concomitant involvement of the mesial temporal lobe (amygdala and anterior hippocampus) and small independent IEDs were also recorded in isolation in the mesial temporal lobe.

In patient 5, depth electrodes showed a predominance of right orbito-frontal and medio-prefrontal IED, concordant with ESIo and the associated BOLD cluster. Much fewer IED were observed in the right supplementary motor area corresponding to the maximum positive BOLD cluster.

In patient 8, depth electrodes showed IED originating from the mesial fronto-parietal cortex (para-central lobule and mesial superior parietal lobe), confirming the agreement between ESIo and the BOLD response.

### Illustrative cases

**Fig. 1:**
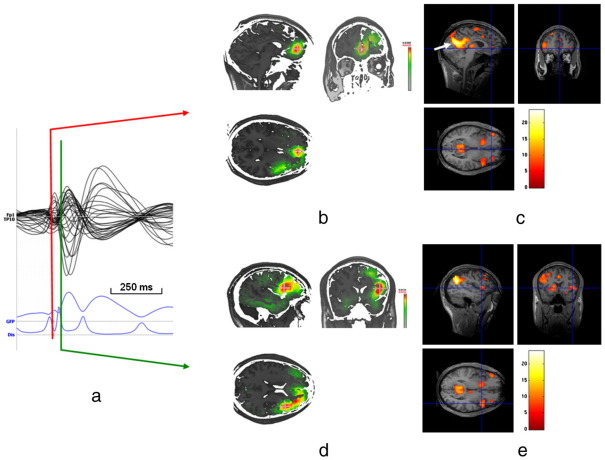
Patient 5: Example of mesial frontal onset with lateral propagation. ESI and EEG-fMRI SPM-F map (canonical HRF and 2 derivatives, Family Wise Error correction for multiple voxel comparisons) overlaid on co-registered T1-weighted image; a) averaged intra-MR IED. The first rising phase of the averaged IED and Global Field Power (GFP) is used for IED onset (ESIo, red line) and a later timeframe for IED propagation (ESIp, + 88 ms, second rising phase of the averaged IED, green line). Dis = Dissimilarity is a measure inversely related to the spatial correlation between two scalp voltage map topographies (not shown): a minimum of Dis therefore reflects a period of map stability (Lantz et al., 2003; Michel et al., 2004); b) EEG source imaging at IED onset (ESIo) in orbito-frontal cortex (bilateral but maximum in left hemisphere); c) right orbito-frontal BOLD cluster concordant to ESIo (positive BOLD response, cross-line at maximum). The highly significant BOLD response in the mesial parietal cortex corresponds to negative BOLD response in the “default mode” network (white arrow); d) EEG source imaging just after second maximum of the averaged IED showing a shift of maximal source activity to frontal-opercular region (ESIp); e) right lateral frontal BOLD cluster closest to ESIp (positive BOLD response, cross-line at maximum).

**Fig. 2:**
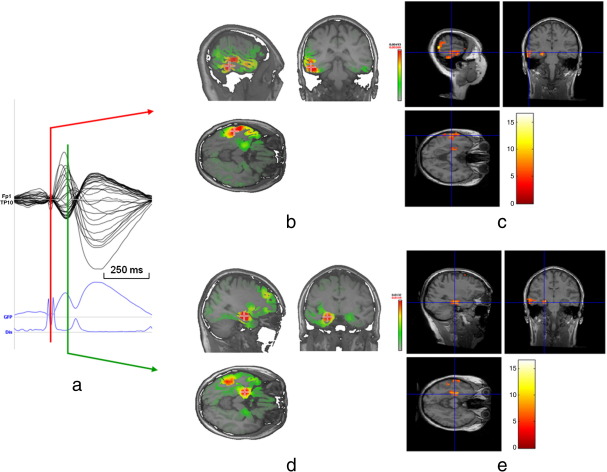
Patient 2: Example of ESIo concordant with negative BOLD response. ESI and EEG-fMRI SPM-F map (canonical HRF and 2 derivatives, FWE corrected) overlaid on co-registered T1-weighted image; a) averaged intra-MR IED. The first rising phase of the averaged IED and Global Field Power (GFP) is used for IED onset (ESIo, red line) and a later timeframe for IED propagation (ESIp, + 96 ms, second maximum of the averaged IED, green line). Dis = Dissimilarity (Lantz et al., 2003; Michel et al., 2004); b) EEG source imaging at IED onset (ESIo) in left lateral temporal cortex; c) left lateral temporal BOLD cluster concordant to ESIo (negative BOLD response, cross-line at maximum); d) EEG source imaging of the second maximum phase of the averaged IED showing a shift of maximal source activity to left mesial temporal lobe (ESIp); e) left mesial temporal BOLD cluster concordant with ESIp (positive BOLD response, cross-line at maximum).

Fig. 1 shows an example of concordance (patient 5, right frontal IED). Here, the combination of ESI and EEG–fMRI allows to identify the orbito-frontal BOLD cluster closest to the irritative zone, and reveal temporal patterns suggestive of propagation from mesial to lateral frontal cortex, subsequently confirmed by intracranial recording. However, the maximum positive BOLD cluster (supplementary motor area) did not correspond to the areas principally involved in IED generation. The most significant voxel showing a BOLD decrease in the retrosplenial region is consistent with previous reports of IED-related BOLD changes in cortical regions involved in the “default mode” network (Kobayashi et al., 2006a; Laufs et al., 2007),

Fig. 2 illustrates patient 3 (left temporal IED) with a negative BOLD cluster as the closest cluster to IEDo (lateral temporal), and subsequent propagation closest to a positive BOLD cluster (mesial temporal). The most significant cluster of positive BOLD response is in the left insular cortex. As in patient 5, the maximal negative BOLD response is in regions typical of the default mode network (not seen on the slice presented in this figure). Both positive and negative maxima are discordant with ESI.

**Fig. 3:**
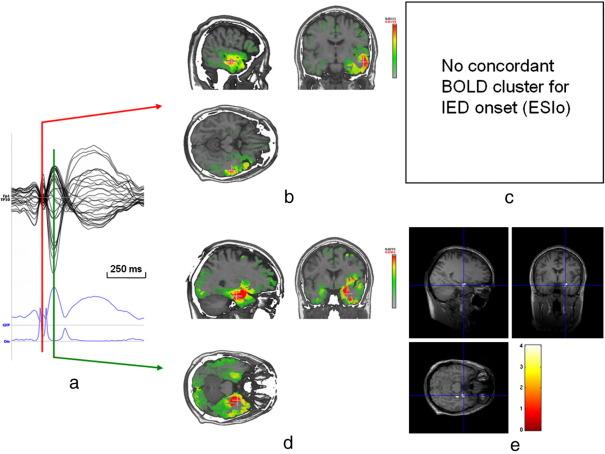
Patient 1: Example of discordant ESIo and EEG-fMRI results. ESI and EEG-fMRI SPM-F map (canonical HRF and 2 derivatives, FWE corrected) overlaid on co-registered T1-weighted image; a) averaged intra-MR IED of the most frequent IED (right temporal). The first rising phase of the averaged IED and Global Field Power (GFP) is used for IED onset (ESIo, red line) and a later timeframe for IED propagation (ESIp, +80 ms, second maximum of the averaged IED, green line). Dis = Dissimilarity, (Lantz et al., 2003; Michel et al., 2004); b) EEG source imaging at IED onset (ESIo) in right lateral temporal cortex; c) there is no concordant mesial temporal BOLD response, even for aggregated IEDs (right temporal, right posterior temporal, right temporo-occipital); d) EEG source imaging of the next maximum of the averaged IED showing a shift of maximal source activity to right mesial temporal lobe (ESIp); e) right mesial temporal BOLD cluster concordant with ESIp (positive BOLD response for aggregated IEDs, cross-line at maximum).

Fig. 3 shows discordant results for ESIo and the BOLD response in patient 1 (combined right temporal IEDs). ESIo of case 1a shows a right lateral temporal activity that propagates to right mesial temporal regions. There is no concordant BOLD response for ESIo in the lateral temporal cortex. However, there is a right mesial temporal positive BOLD response concordant with ESIp. Intracranial recording confirmed IED originating from the right mid- to posterior temporal neocortex.

## Discussion

The purpose of this study was to use ESI in order to extract more information from the temporally blurred BOLD patterns revealed by EEG–fMRI analysis. In this respect, the temporal resolution of ESI and its feasibility on the same dataset seems an ideal combination. Our aim was not to assess whether ESI or fMRI was better at localising the epileptic focus with respect to intracranial EEG recording as a gold standard. Good concordance between ESI, EEG–fMRI and subdural grid EEG recording in a single patient has been reported in an early EEG–fMRI paper (Seeck et al., 1998) and, more recently, a study of 5 patients with focal epilepsy concluded that the results of ESI using multiple equivalent dipoles and clusters of significant BOLD responses were concordant both with each other and with intracranial recording (Benar et al., 2006). Another study using a distributed inverse solution also showed good concordance between both modalities and intracranial EEG recordings in 3 patients with focal epilepsy (Grova et al., 2008). Concordance between ESI and BOLD increases confidence in both modalities and therefore in the localising information they provide. In our study, intracranial EEG recordings confirmed the spatio-temporal pattern of propagation in the 3 patients for which they were available.

Our study compared BOLD correlates of IED to electric source imaging performed on the same set of IED acquired inside the MR scanner. This represents an important step when comparing non-invasive modalities and opens the possibility of studying single events, as has previously been applied to event-related potentials (Debener et al., 2005). Moreover the analysis of simultaneously acquired data eliminates bias associated with separate single modality sessions, such as the extent and propagation of IED which may be altered by experimental conditions and clinical context (degree of arousal, medication, time since last seizure) for example. This is particularly relevant in epilepsy where spontaneous IED generation cannot be identically reproduced (Liu et al., 2006). Using the same set of IED for both analyses is methodologically stringent from the viewpoint of the comparison of the results of both modalities but this is not standard practice for ESI, as studies usually include only selected spikes with good signal to noise ratio. Previous studies evaluated the concordance of ESI and BOLD responses to IED in focal epilepsy using different ESI methodology but always acquired the EEG recording for the ESI in a separate session outside the scanner, generally with additional electrode coverage (Lemieux et al., 2001; Benar et al., 2006; Boor et al., 2007; Grova et al., 2008). Our study shows that MR-related EEG artefacts and the application of correction algorithms do not preclude ESI on intra-MR EEG. This is consistent with previous studies which reported only minor distortion of IED after EEG correction, compared to outside the scanner environment (Benar et al., 2002; Benar et al., 2003; Salek-Haddadi et al., 2003; Salek-Haddadi et al., 2006).

### Concordance and sources of uncertainties

Perfect overlap between fMRI and ESI results is not expected due to the different nature of the two signals: EEG arises from the sum of synchronised post-synaptic activity while BOLD response originates from haemodynamic changes related to total synaptic activity, this signal being partly localised at the site of metabolic change and partly in distally draining veins, the latter effect being dependent on the scanner field strength (Logothetis et al., 2001; Turner, 2002). Additionally, changes in EEG and BOLD signal might be caused by different cellular populations which might or might not overlap spatially (Nunez and Silberstein, 2000). Experimental measurements on the sensory cortex of monkeys has shown an average distance of 10 mm between activated subdural micro-electrodes and fRMI centroids in 45% of observations (Disbrow et al., 2000). In this work, we found good agreement when measuring distances between peak local field potential, estimated with ESI, and the maximal activated voxel in the nearest BOLD cluster in order to quantify the spatial concordance between the two techniques. The possible sources of error in our ESI analysis include the ill-posed nature of the inverse problem, the use of standardised electrode positions (based on the 10–20 electrode position system), a sampling distance of 4–6 mm between solution points and the bias towards superficial sources. Regarding fMRI data, distortion and drop-out of fMRI signal especially at air–tissue interfaces, smoothing of fMRI data and coregistration of anatomical and EPI images can lead to degradation in sensitivity and localisation accuracy. Furthermore, the varying number of spikes captured during the EEG–fMRI recordings is an additional source of uncertainty, which affects both modalities equally but makes inter-subject comparisons more difficult.

All the above factors contribute to the inter-subject variability of the peak-to-peak distance observed in our study. It must be emphasized, however, that epileptic activity can never be pinpointed to a cortical region at a microscopic level because it involves the participation of large neuronal networks. IED detectable on the scalp EEG and hence modelled with either ESI or EEG–fMRI require the involvement of at least 6 cm^2^ of cortex (Tao et al., 2007). This argues in favour of our choice of a sub-lobar scale for determining spatial concordance between the electro-clinical data and the two imaging techniques. Our spatial concordance is better than that reported in previous studies comparing dipole models of ESI with clusters of BOLD changes correlated to IEDs (Lemieux et al., 2001; Bagshaw et al., 2006). This adds evidence to the argument that distributed inverse solutions are better suited as a model of the widespread cortical activity such as involved in the onset and propagation of IEDs (Michel et al., 2004; Liu et al., 2006) Nevertheless, further studies are needed to determine the origin of the discrepancies between ESI and BOLD in epilepsy, and how they relate to factors such as cortical location.

### ESI and EEG–fMRI of IED in the mesial cortex

In the cases with a mesial frontal epileptogenic focus (patients 5 and 8), we found that the ESI maximum was lateralised to the contra-lateral hemisphere whereas the BOLD responses were concordant with other electro-clinical data. False lateralisation of midline activity with ESI can arise due to slight displacement of the electrode cap or topography of activated gyri and sulci (Michel et al., 2004). Although these results have to be considered discordant in view of our criteria, they still provide valuable information to help interpret the multiple BOLD clusters, because despite limited spatial accuracy they indicate a mesial frontal IED focus. Equally, the strongly lateralised, spatially well defined EEG–fMRI results, concordant with electro-clinical information, were helpful to correct the mis-lateralisation of ESI results further highlighting the two techniques complementarities.

### ESI and EEG–fMRI in temporal lobe IED

The two other discordant cases (studies 1 and 4) illustrate the different methodological limitations affecting EEG–fMRI and ESI in patients with temporal lobe IED and thereby also illustrate the advantage of combining these imaging techniques for a better understanding of the underlying disease. Patient 1 showed a right occipito-temporal neocortical BOLD change corresponding to ESIo for onset of IED, but there was no significant BOLD change linked to the other 2 sets of IED. In a secondary analysis, we combined all three types of temporal IEDs thereby potentially increasing sensitivity if the three event types share a common haemodynamic substrate (Salek-Haddadi et al., 2006). The resulting map revealed a significant hippocampal response that was concordant with ESIp. Taken together, this suggests that in the case of a widespread irritative zone, the mesial temporal structures are a common denominator in the epileptic network of each distinct IED type; a result confirmed by intracranial EEG in this case. This is in line with previous group analysis of patients with temporal lobe epilepsy using EEG–fMRI (Laufs et al., 2007).

In patient 4 (left mesial occipital cortical dysplasia with left and right temporal IED) only a very small cluster of significant voxels was obtained for the less frequent right temporal spikes whereas the more numerous left temporal IED had no associated significant BOLD change. There was also no significant corrected BOLD signal change in the lesional occipital lobe. Possible explanations for this result include signal drop-out in the basal temporal lobe limits sensitivity to BOLD signal changes in this location (Bagshaw et al., 2004). Moreover, insufficient EEG electrode coverage, especially of the lower temporal regions can limit ESI localisation (Lantz et al., 2003a; Sperli et al., 2006). Despite these limitations, our findings are consistent with intracranial studies of epileptic networks in patients with temporal lobe epilepsy, that describe a strong interaction of the epileptogenic activity in mesial temporal and neocortical regions (Bartolomei et al., 2001). While some authors consider that ESI is not able to recover isolated temporal mesial activity (Ebersole, 1997; Merlet and Gotman, 2001; Gavaret et al., 2004) other studies suggested the opposite, at least when the entorhinal cortex was involved (Lantz et al., 1997; Pacia and Ebersole, 1997; Zumsteg et al., 2006; James et al., 2008). Previous EEG–fMRI studies focusing on patients with temporal lobe epilepsy showed only rare temporal mesial BOLD responses despite the presence of hippocampal atrophy in several patients (Kobayashi et al., 2006a; Laufs et al., 2007). BOLD responses were also found in the contralateral temporal lobe and extra-temporal areas, such as the peri-insular/opercular cortex (as in patients 1 and 2). Further studies, involving better lower temporal electrode coverage and systematic intracranial validation, are necessary to assess the usefulness of EEG–fMRI to solve this ESI dilemma and help determine whether the combined methods can help discriminate between mesial and lateral temporal epileptogenic foci.

### ESI as a marker of propagation

When the two techniques give concordant localisation, the higher temporal resolution of ESI may be used to attempt to discriminate between BOLD clusters related to early vs. late IED components and provide dynamic information about the network. The rising phase of IED has been shown to be the best estimate for the localisation of IED onset, while source activity related to later timeframes indicates propagation (Lantz et al., 2003b; Ray et al., 2007). In our study, when ESI showed propagation, this second region of maximal local field potential was related to a second concordant BOLD cluster in 6/12 cases. It remains unclear why the remaining cases showed no significant BOLD cluster concordant with ESIp although, in 2/6 of these, a BOLD cluster concordant with ESI was observed in data uncorrected for multiple comparisons. This could reflect an inadequate model of the BOLD signal due to lack of IED, poor representation of the IED-related BOLD time course by the basis set of the HRF chosen in this study, although significant deviations are probably rare (Salek-Haddadi et al., 2006; Lemieux et al., 2008) or poor representation of the baseline (Salek-Haddadi et al., 2003). There was no clear cut difference in the number of IED for studies with BOLD cluster concordant with ESIp compared to those without. The localisation and propagation patterns revealed by intracranial EEG in 3 patients were in line with the ESI results. While this increases confidence in ESI-defined propagation in the other patients without intracranial recording, it is not possible to be sure if incorrect ESI or a lack of sensitivity of EEG–fMRI is the reason for any mismatch.

### Revisiting the importance of the cluster containing the most significant BOLD increase

Previous EEG–fMRI studies have analysed EEG–fMRI results with particular attention paid to the maximal significant positive BOLD cluster in the hope of identifying a unique marker of epileptogenicity (Salek-Haddadi et al., 2006). These authors assessed concordance at a lobar level between BOLD response at 1.5 T and non-invasive clinical data (interictal/ictal EEG, abnormality on structural MRI if available). They showed that the cluster of maximal positive statistical value was generally concordant with the presumed focus at the lobar level. The other, less significant clusters, and in particular those corresponding to BOLD decreases, were suggested to reflect IED propagation or distant activation/deactivation of neuronal networks in response to IEDs. In this study, we found that the BOLD cluster containing the global statistical maximum was often not the closest to the region of IED onset as identified by ESIo. In one case, intracranial EEG confirmed that a BOLD cluster other than that containing the global maximum was the main IED generator (patient 5). If ESI had not suggested this region for IED onset, the concordant very small cluster of positive BOLD change may have been overlooked. Therefore, even very small clusters of BOLD change should be a priori considered as potential candidates for localising the IED generator. In this case, the most significant positive and negative BOLD responses (supplementary motor cortex and precuneus, respectively) were distant from the intracranial spiking electrode contacts. However, when concordance is assessed only at a lobar level, the maximal positive BOLD cluster and ESIo are found to be concordant found in 5 additional cases (cases 1abc, 2, 3, 6a, 7a) increasing concordance to 75% (9/12) cases, which is similar to the 74% (17/23) value in Salek-Haddadi et al. (2006). The finding that the most significant BOLD cluster does not reliably indicate the primary generator of IEDs is consistent with ictal SPECT studies, where the area of maximal perfusion usually corresponds to the area of seizure propagation rather than to the ictal onset zone (Van Paesschen et al., 2007).

### ESI and negative BOLD responses

In some cases, we found that the closest BOLD cluster to ESI results corresponded to a negative BOLD change. Negative IED-related BOLD changes have been increasingly reported both close to and distant from epileptogenic foci (Kobayashi et al., 2006b; Salek-Haddadi et al., 2006; Jacobs et al., 2007). They have been attributed to a decrease in metabolic demand (deactivation), relying on the assumption that neurovascular coupling is maintained in the irritative zone both during baseline and IED generation, for which there is some limited evidence mainly in relation to generalised discharges (Stefanovic et al., 2005; Carmichael et al., 2008; Hamandi et al., 2008). Whereas distant negative BOLD changes have generally been attributed to IED-induced changes in the brain resting state, BOLD decreases local to the presumed focus are more difficult to interpret. Some authors have suggested a local vascular steal or surround neuronal inhibition with low metabolic demand as possible mechanisms (Shmuel et al., 2002; Bagshaw et al., 2004). Decrease in excitatory input (“deactivation”) has been related to BOLD decreases whereas neuronal inhibitory activity is intrinsically a metabolically demanding process associated with increase of the BOLD signal (Lauritzen and Gold, 2003). Despite findings in animals and humans that a negative BOLD response was associated with decreased neuronal activity during a visual task (Shmuel et al., 2002; Shmuel et al., 2006) this is not necessarily the case for epileptic activity. First, highly synchronised oscillations responsible for epileptic discharges seen on the EEG are not necessary paired with regional increases of total synaptic activity. Concurrent decreases of the rest of the network activity (or decreased inhibitory activity) could result in negative BOLD response caused by a net decreased metabolism, as also seen with interictal FDG-Positron Emission Tomography (Van Paesschen et al., 2007). MR perfusion studies of IED-related perfusion changes are difficult to conduct since a high rate of IED is needed due to the low signal-to-noise ratio of perfusion imaging. Second, fMRI and optical imaging studies have revealed a mismatch between brain oxygenation and perfusion in animal models of epilepsy (Bahar et al., 2006; Schridde et al., 2008) which as also been reported in intra-operative recording of neocortical seizures in the human cortex (Zhao et al., 2007). Therefore, BOLD negative responses could occur even if total synaptic activity and metabolic demand are increased. Moreover, intracranial electrodes have recorded epileptogenic activity close to negative BOLD responses (Benar et al., 2006).

ESIo closest to regions of BOLD decrease were found predominantly in the temporal lobe in our study. In 2 cases that exhibited only BOLD decreases, we did not find positive BOLD changes with concordant anatomic localisation and in the 2 other cases positive BOLD changes were located at much greater distance. This suggests that a potential ESI inaccuracy is not sufficient to explain our findings. In patient 2, there was interplay between right mesial and lateral temporal activity, with both regions being active at onset and maximum activation, switching from lateral to mesial structures. Even if we reason that the true IED onset zone were in reality closer to the cluster of BOLD increase located in the mesial temporal region, it would still be important to understand the pathological significance of BOLD decreases in the region of lateral temporal propagation which generated IED detected on the scalp EEG.

Therefore, both positive and negative BOLD changes should be considered when evaluating epileptic networks with EEG–fMRI, especially in the context of pre-surgical evaluation. A greater number of patients with intracranial EEG recording is needed to confirm the significance of “secondary” clusters of BOLD increase or decrease concordant with ESI localisation.

### Conclusion

In conclusion, simultaneous ESI and fMRI appears to be a methodologically robust way of combining both modalities, which can provide new information on the dynamics of epileptic networks. It can be used to provide temporal differentiation of the often complex patterns of IED-related BOLD changes that are encountered with EEG–fMRI studies, and in particular to increase confidence in the location of the primary focus when spatially concordant. The high temporal resolution of ESI can improve the localising value of EEG–fMRI, while the spatial definition provided by the BOLD clusters may increase confidence in the ESI localisation. This synergy could improve the clinical decision making, for instance when defining regions of interest for intracranial EEG recording.

## Figures and Tables

**Table 1:** Clinical, electrophysiological and imaging data

Case	Gender, age	Seizure semiology	IED localisation on scalp EEG used for fMRI modelling	Number of IED used for fMRI modelling (Number of 20 min fMRI sessions)	MRI
1a	M, 31 y.	Bizzare vague sensation, CPS with dystonic posture L hand	R mid temporal	103 (2)^a^	N
1b	R post temporal	17 (2)^a^
1c	R occipito-temporal	69 (2)
2	M, 30 y.	Aphasic SPS No aura, CPS	L temporal	161 (2)	N
3	F, 48 y.	No aura, CPS with oral and manual automatisms	L temporal	197 (2)	N
4a	M, 21 y.	CPS, oral automatisms, R clonic jerks, SGS	L temporal	312 (2)^a^	FCD L mesial occipital
4b	R temporal	56 (2)
5	M, 27 y.	Cloni R face/arm → SGS	R frontal	2239 (2)	N
6a	M, 22 y.	No aura, CPS with L head version	Bil frontal	269 (3)	N
6b	Bil frontal polyspikes	101 (3)
7a	F, 37 y.	No aura, CPS	L frontal	23 (2)	N
7b	Bil frontal	49 (2)
7c	R frontal	11 (2) a
8a	M, 19 y.	Epilepsia partialis continua L leg → rare SGS	R fronto-central	121 (3)	N
8b	Central midline	206 (3)
8c	R frontal	10 (3)^a^
9a	M, 25 y.	Epigastric, auditory, gustatory or heautoscopic aura, tonic posture R hand	L fronto-temporal	38 (3)	N
9b	L fronto-polar	35 (3)^a^
9c	L parietal	23 (3)^a^

Clinical, EEG and radiological data. R/L/Bil: right/left/bilateral; post: posterior, SPS/CPS: Simple/Complex Partial Seizure, SGS: Secondarily Generalised Tonic Clonic Seizure, FCD: Focal Cortical Dysplasia.

a No significant IED-related BOLD change revealed and not considered individually further for ESI.

**Table 2:** EEG source imaging and EEG-correlated fMRI results

	EEG focus (total IED)	Main BOLD+ cluster (*F*-test, FWE)	ESI onset	Closest BOLD (mm) (*F*-test, FWE)		ESI propagation	Closest BOLD (mm) (*F*-test, FWE)		Intracranial EEG IED
P1c	R occipito-temporal (69)	Only BOLD neg R occipito-temporal	R occipito-temporal	−	25	Idem	+	19	R lat mid- and post temporal
P1a	R mid-temp	Joint analysis: R ant cingulate	R lat mid-temp	NA (+ unc)	NA	R mes temporal	+	10	“
P1b	R post temp	R post-temp	–
P1c	R occip-temp (total 189)	R occip-temp	–
P2	L temporal (161)	L inf temporo-occipital	L lat temporal	−	19	L mes temporal	NA	NA	
+ (max)	51
P3	L temporal (197)	L insula and L opercular frontal	L lat temporal	−	6	L mes temporal	+	6	
+	30
P4b	R temporal (56)	R temporo-parietal (unc: R insula, R inf lat temporal)	R lat ant temporal	NA	NA	R mid frontal	NA	NA	
P5	R frontal (2239)	R > L mes sup frontal	L > R mesial orbito-frontal	+ contralat (max)	10	R lat inf frontal	+	17	R mesio-orbito-frontal and mesial prefrontal
P6a	Bil frontal (269)	R sup frontal	R ant frontal	+	16	L mes frontal	+	25	
P6b	Bil frontal polyspikes	R sup frontal	NA			NA			
P7a	L frontal (23)	L mid frontal	L inf frontal	+	32	L mid frontal	+	11	
P7b	Bil frontal (49)	L mid frontal	L mid frontal	+ (max)	19	R sup frontal	NA (+ unc)	NA	
P8a	R fronto-central (121)	R mid frontal (R sup frontal)	R sup frontal	+ (max)	9	L mid frontal	NA	NA	R mesial fronto-parietal
P8b	central midline (206)	R frontal midline	L frontal midline	+ contralat (max)	31	R mid frontal	NA (+ unc)	NA	"
P9a	L fronto-temporal (38)	Only BOLD neg: L opercular fronto-temporal	L lat temporal	−	33	L mes temporal	NA	NA	"

EEG–fMRI and ESI concordance: FWE: Family Wise Error correction, unc.: uncorrected; R/L/Bil: Right/Left/Bilateral, ant/post: anterior/posterior, sup/mid/inf: superior/middle/inferior, mes/lat:mesial/lateral. NA: not available.
